# Methyl aminolevulinate photodynamic therapy for Bowen's disease on the fingers—Is monotherapy sufficiently effective?

**DOI:** 10.1111/phpp.12586

**Published:** 2020-07-20

**Authors:** Maria‐Lisa Repelnig, Wolfgang Weger, Urban Cerpes, Peter Wolf, Franz Josef Legat

**Affiliations:** ^1^ Department of Dermatology and Venerology Medical University of Graz Graz Austria

Bowen’s disease (BD), also known as squamous cell carcinoma (SCC) in situ, most commonly involves the head and neck area as well as the extremities,^1^ but little epidemiological data exists about its prevalence on the fingers. Several treatment modalities are available for BD, including surgical excision, photodynamic therapy (PDT), topical 5‐fluorouracil, imiquimod, and cryotherapy, but no single therapy has been proven to be superior to others.^2^ Several case reports have previously shown that PDT may be an effective treatment for BD on the fingers.^3^, ^4^, ^5^ To evaluate the effectiveness of methyl aminolevulinate (MAL)‐PDT in eradicating BD on the fingers, we retrospectively analysed a case series of six patients with biopsy‐proven BD and one patient with bowenoid hyperplastic actinic keratosis on at least one of their fingers for their response to MAL‐PDT. The analytical methods used in the study were performed in accordance with ethical approval no. 25‐294 ex 12/13 of the Ethics Committee of the Medical University of Graz, Graz, Austria.

Seven patients (four women and three men; median age 77 years, ranging from 41 to 88 years) were treated with MAL‐PDT between 2016 and 2019. The outcome was included in the analysis. One man had lesions on two of his fingers (Table 1; no. 4); the other six patients each had one lesion on one of their fingers. Biopsy samples from five patients (1 each in 4 patients and 2 in the patient with 2 lesions) were analysed for the presence of human papillomavirus (HPV) using PCR (Table 1).

**TABLE 1:** Results of photodynamic therapy in patients with Bowen’s disease and bowenoid keratosis

Patient no.	Diagnosis	Sex	Age	Location	Lesion size (cm × cm)	HPV PCR results	Number of PDT sessions	Time frame of PDT treatment (weeks)	Response	Previous treatment
1	BD	m	41	Distal phalanx left index finger	2.5 × 2	HPV 16	4	5	No	None
2	BD	m	55	Distal phalanx left middle finger	2.5 × 2	HPV 16	10	53	Partial	Shaving
3	BD	f	77	Distal phalanx left little finger	1 × 1	Not examined	6	75	Partial	None
4	BD	m	88	Proximal phalanx right index finger	0.7 × 0.7	HPV 39	6	23	Partial	Topical diclofenac 3% gel
				proximal phalanx left index finger	1 × 0.5	Undetectable	6	23	Partial	Topical diclofenac 3% gel
5	BD	f	78	Proximal phalanx left index finger	2 × 0.5	HPV 16	2	1	Complete	Shaving
6	BD	f	73	middle phalanx left ring finger	2.5 × 0.5	not examined	2	1	Partial	None
7	Hyperplastic bowenoid actinic keratosis	f	81	Proximal phalanx right index finger	4 × 3	Undetectable	4	4	No	Shaving, imiquimod 5% cream, ingenol mebutate gel, 5‐fluorouracil 0.5% solution

HPV16 was found in the BD lesions of three patients (Table 1; nos. 1, 2, and 5), and HPV39 was found in one BD lesion of a patient (Table 1; no. 4).

The seven patients received a median number of 4 MAL‐PDT sessions (2‐10 sessions) over a median timeframe of 5 weeks (1‐75 weeks). On the treatment days, MAL cream was applied to the skin lesions after superficial curettage was performed; lesions were then occluded with a light‐impermeable wound dressing for 3 hours. Afterwards, the wound dressing was removed, and excess MAL cream was wiped off. Immediately thereafter, the lesion was irradiated with red light (Aktilite LED lamp, 630 nm, 37 J/cm^2^). This treatment was performed at least twice per patient, with one week between treatments.

The treatment response was evaluated at a median of 10 weeks (8‐18 weeks) after the last PDT as no, partial, or complete response. This response was evaluated clinically by a physician experienced in PDT using images and clinical descriptions of the lesions. PDT‐treated BD lesions showed a partial response in 4 patients, and no response in 2 patients. Only 1 of the patients (Table 1; no. 7) showed clearance of BD after PDT.

HPV16 is the most common HPV subtype identified in SCC. Detection of the same HPV types in the anogenital lesions and the BD of the nail unit suggests the possibility of auto‐inoculation of the finger with HPV from the anogenital area.^6^, ^7^ In our case series, only one HPV16‐positive patient had a history of genital condyloma removal 30 years ago. In previous studies, the metastasis rates in digital SCC did not depend on HPV association, however, the digital invasive SCC and SCC in situ associated with high‐risk HPV appeared to be more locally aggressive.^6^, ^7^ A locally aggressive growth was seen in 1 (Table 1; no. 1) of the 3 HPV16‐positive patients. The HPV39 positive BD lesion (Table 1; no. 4) had a rather regular tumour growth, although HPV39 has been generally considered to be a high‐risk genotype. To the best of our knowledge, HPV39 positivity has not been previously reported in BD. A recent case series indicated that ﻿the presence of HPV16 in acral BD could be a predictor for a reduced response to PDT.^8^ The HPV16‐positive patient in this series, whose BD displayed locally aggressive growth, showed no response to PDT. However, no overall correlation between HPV status and response to PDT was found in this case series.

In conclusion, this retrospective analysis of BD in a small number of patients suggests that conventional monotherapy with MAL‐PDT may not be sufficiently effective to eradicate BD on the fingers, regardless of the patient’s HPV status.

## CONFLICTS OF INTEREST

The authors MR, PW, and FJL received support from Galderma to attend Euro‐PDT‐meetings.
